# Association between *LTA*, *TNF* and *AGER* Polymorphisms and Late Diabetic Complications

**DOI:** 10.1371/journal.pone.0002546

**Published:** 2008-06-25

**Authors:** Eero Lindholm, Ekaterina Bakhtadze, Corrado Cilio, Elisabet Agardh, Leif Groop, Carl-David Agardh

**Affiliations:** Department of Clinical Sciences, University Hospital MAS, Lund University, Lund, Sweden; NICHD/National Institutes of Health, United States of America

## Abstract

**Background:**

Several candidate genes on the short arm of chromosome 6 including the *HLA* locus, *TNF*, *LTA* and *AGER* could be associated with late diabetic complications. The aim of our study was therefore to explore whether polymorphisms (*TNF* -308 G→A, *LTA* T60N C→A and *AGER* -374 T→A) in these genes alone or together (as haplotypes) increased the risk for diabetic complications.

**Methodology/Principal Findings:**

The studied polymorphisms were genotyped in 742 type 1 and 2957 type 2 diabetic patients as well as in 206 non-diabetic control subjects. The Haploview program was used to analyze putative linkage disequilibrium between studied polymorphisms. The *TNF*, *LTA* and *AGER* polymorphisms were associated with the *HLA-DQB1* risk genotypes. The *AGER* -374 A allele was more common in type 1 diabetic patients with than without diabetic nephropathy (31.2 vs. 28.4%, p = 0.007). In a logistic regression analysis, the *LTA* but not the *AGER* polymorphism was associated with diabetic nephropathy (OR 2.55[1.11–5.86], p = 0.03). The *AGER* -374 A allele was associated with increased risk of sight threatening retinopathy in type 2 diabetic patients (1.65[1.11–2.45], p = 0.01) and also with increased risk for macrovascular disease in type 1 diabetic patients (OR 2.05[1.19–3.54], p = 0.01), but with decreased risk for macrovascular disease in type 2 diabetic patients (OR 0.66[0.49–0.90], p = 0.009). The *TNF* A allele was associated with increased risk for macrovascular complications in type 2 (OR 1.53 [1.04–2.25], p = 0.03, but not in type 1 diabetic patients.

**Conclusions/Significance:**

The association between diabetic complications and *LTA*, *TNF* and *AGER* polymorphisms is complex, with partly different alleles conferring susceptibility in type 1 and type 2 diabetic patients. We can not exclude the possibility that the genes are part of a large haplotype block that also includes *HLA-DQB1* risk genotypes.

## Introduction

The etiology of diabetic complications is complex, and inflammation may play a role [1]. The mRNA expression for pro-inflammatory cytokines such as IL-1 and Tumor Necrosis Factor Alpha (TNF-α) is increased in the retina and animal studies suggest that inhibition of TNF-α has beneficial effects in prevention of diabetic retinopathy [2], [3]. Recently, we have shown that type 1 diabetic patients with proliferative retinopathy have increased levels of TNF- α [4]. Similarly, inflammatory markers are elevated in diabetic nephropathy [5] and inflammation is associated with development of macrovascular complications such as myocardial infarction [6]. TNF-α and lymphotoxin-α (LT-α, also known as TNF-β) belong to the same TNF family and are encoded by the same gene cluster. TNF-α is mainly produced by activated macrophages and LT-α by T, B and natural killer cell lymphocytes [7]. Promoter variants -308A→G and -238G→A in the gene coding for TNF-α (*TNF*) affect transcriptional regulation of the gene coding for LT-α (*LTA*) [8]. The receptor for advanced glycation end products (RAGE) is also mainly considered as an intracellular signal and transducer or proinflammatory peptide [9].

*LTA*, *TNF* and the gene encoding for RAGE (*AGER*) are all located within the MHC complex on the short arm of chromosome 6. The HLA locus is among the most polymorphic in the human genome. Some studies have suggested a direct role of HLA in development of diabetic nephropathy [10], retinopathy [11] and macrovascular disease [12]. Since this region also harbours a variety of other genes involved in inflammation, the results could also reflect variation in other genes than HLA. We have recently shown *AGER* -374T→A polymorphism to be associated with diabetic nephropathy and possibly with retinopathy in type 1, but not in type 2 diabetic patients [13]. The results concerning the risk allele (A) in the *AGER* gene were in conflict with a previous study [14], and a possible explanation could be that the *AGER* gene is in linkage disequilibrium with other genes, such as *TNF* and *LTA*.

Variation in *TNF* and *LTA* genes has been associated with diabetic nephropathy [15], retinopathy [16] as well as with cardiovascular and cerebrovascular disease [17], [18]. A large genome-scan in Japanese patients identified a susceptibility locus for myocardial infarction on chromosome 6p21 [19], especially the 256A→G and T60N (also referred to as T26N in some studies) variants in the *LTA* gene, were associated with myocardial infarction. However, a recent, rather large study from USA could neither confirm the association with myocardial infarction, nor the association with inflammatory biomarkers [20].

The *TNF*/*LTA* locus is in linkage disequilibrium with *HLA-DQB1* [21] and we have previously shown that the *AGER* -374T→A polymorphism is associated with the *HLA-DQB1* risk genotypes [13]. In a recent study the HLA 8.1 ancestral haplotype was shown to be strongly linked to the C allele of the *AGER* -429T→C promoter polymorphism [22]. Our aim was to study whether variants in these genes form a putative haplotype associated with increased risk of diabetic nephropathy, retinopathy and macrovascular disease.

## Results

Type 2 diabetic patients were older and had higher BMI than type 1 diabetic patients or non-diabetic controls (Table 1). Genotype distributions of *LTA*, *TNF* and *AGER* polymorphisms are shown in Table 2. All three variants deviated from the Hardy–Weinberg equilibrium in type 1, but neither in type 2 diabetic patients nor in non-diabetic controls. In type 1 diabetic patients there was an excess of heterozygous patients with *LTA* (p<0.0002), *TNF* (p = 0.02) and *AGER* (p = 0.008). The genotype frequencies of the *LTA*, *TNF* and *AGER* polymorphisms were different between type 1 diabetic patients and controls and also between type 1 and type 2 diabetic patients. The *LTA*, *TNF* and *AGER* polymorphisms were associated with the *HLA-DQB1* genotypes (Table 3). The minor allele (A) of the *LTA* polymorphism was less common in patients with than without *HLA-DQB1* genotypes risk genotypes (37.2 vs. 43.9%, p = 0.0001). No difference in allele frequencies of the *TNF* polymorphism was seen between patients with or without *HLA-DQB1* risk genotypes, however the minor allele (A) of the *AGER* polymorphism was more common in patients with than without *HLA-DQB1* risk alleles (34.5 vs. 22.9%), p<0.000001). Because of lack of Hardy–Weinberg equilibrium in type 1 diabetic patients, we could not confirm whether the *LTA*, *TNF* and *AGER* polymorphisms were in LD in type 1 diabetic patients. In type 2 diabetic patients and controls the LTA and *TNF* polymorphisms were in LD (D′ = 1.00 [0.99–1.00] , r^2^ = 0.4). The *AGER* polymorphism was neither in LD with *LTA* (D′ 0.13 [0.06–0.20], r^2^ = 0.004) nor with *TNF* (D′ 0.36 [0.23–0.43], r^2^ = 0.009) in type 2 diabetic patients.

**Table 1:** Clinical characteristics of the patients and non-diabetic control subjects.

	Controls	Type 1	Type 2
N (M/F)	107/99	458/375	2240/1616
Age (yrs.)	59.7±12.6^a^	38.2±13.6	61.2±11.7^b^
Age at diagnosis (yrs.)	-	18.2±9.1	55.6±12.2
Diabetes duration (yrs.)	-	18.1[8.5–29.6]	2.58[0.09–9.54]
BMI (kg/m^2^)	25.8±3.8^a^	23.8±3.1	29.7±5.6^a^
HbA_1c_ (%)	-	7.2±1.3	6.6±1.3
Systolic BP (mmHg)	-	130.6±18.9	144.8±21.8
Diastolic BP (mmHg)	-	74.2±9.6	80.9±10.9
Smokers (current or previous)	-	51.5%	61.1%

a p<0.000001.

b p = 0.01. Type 1 and type 2 diabetic patients vs. controls.

**Table 2:** Genotype frequencies (%) of *LTA* T60N (C→A), *TNF* -308 G→A and *AGER* -374 T→A polymorphism in nondiabetic controls and in type 1 and type 2 diabetic patients.

	Controls	Type 1	p	Corrected p	Type 2		Corrected p
*LTA* T60N (CC/CA/AA)	77/88/35 (38.5/44.0/17.5)	186/409/131 (25.6/56.3/18.0)	0.0003	0.05	1089/1395/436 (37.3/47.8/14.9)	0.73	1.00
*TNF* -308 (GG/GA/AA)	133/66/6 (64.9/32.2/2.9)	350/329/50 (48.0/45.1/6.9)	0.00002	0.00009	1908/906/113 (65.2/31.0/3.9)	0.93	1.00
*AGER* -374 (TT/TA/AA)	127/67/11 (62.0/32.7/5.4)	350/335/48 (47.7/45.7/6.5)	0.0003	0.007	1624/1108/198 (55.4/37.8/6.8)	0.07	0.31

The uncorrected p-value refers to differences in genotype frequencies (CC vs CA/AA, GG vs. GA/AA and TT vs. TA/AA). Corrected p-value refers to differences in allele frequencies after 100 000 permutations (haploview).

**Table 3:** Minor allele frequencies according to HLA-DQB1 genotype in type 1 diabetic patients.

	LTA (A allele)^a^	TNF (A allele)^b^	AGER (A allele)^c^
02/0301	26 (59.1)	17 (38.6)	5 (11.9)
02/0302	214 (46.7)	174 (38.2)	131 (28.0)
02/0602^d^	2 (100.0)	1 (50.0)	1 (50.0)
02/0603^d^	6 (50.0)	5 (41.7)	2 (16.7)
02/0604	26 (68.4)	13 (34.2)	2 (5.0)
02/X	114 (67.1)	101 (58.7)	20 (11.6)
0301/0302	29 (45.3)	13 (20.3)	28 (43.8)
0301/0602^d^	1 (50.0)	0 (0.0)	2 (100.0)
0301/0603^d^	5 (83.3)	0 (0.0)	1 (16.7)
0301/0604^d^	3 (50.0)	0 (0.0)	1 (16.7)
0301/X	9 (40.9)	0 (0.0)	9 (40.9)
0302/0602^d^	1 (16.7)	0 (0.0)	3 (37.5)
0302/0603	33 (43.4)	6 (20.0)	14 (46.7)
0302/0604	33 (43.4)	4 (5.1)	20 (26.3)
0302/X	54 (22.7)	18 (7.5)	116 (48.3)
0602/03/04/X^d^	8 (42.9)	12 (14.3)	11 (21.4)
0604/X	10 (50.0)	1 (5.0)	5 (25.0)
X/X	9 (45.0)	2 (10.0)	8 (40.0)

Numbers are number of alleles N (%).

a P<0.000001, Chi-Square = 94.2 df = 11.

b P<0.000001, Chi-Square = 196.3,df = 11.

c P<0.000001, Chi-Square = 98.8,df = 11.

d Expected value <5 in type 1 diabetes. These genotypes were pooled in the statistical analysis. X could mean either a homozygous allele or any allele other than 02, 0301, 0302, 0602 or 0604.

### Diabetic Nephropathy

**Figure 1:**
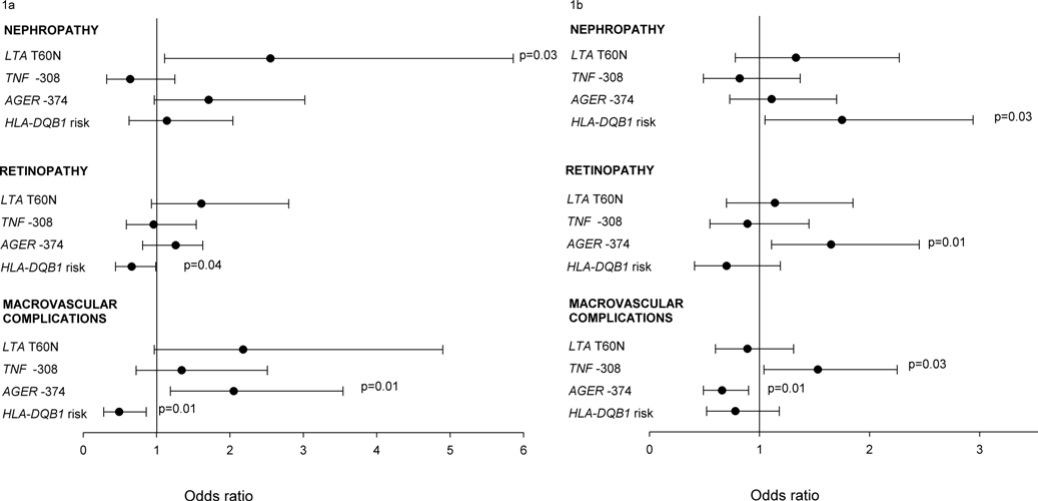
Logistic regression analysis in type 1 (1a) and type 2 (1b) diabetic patients with *LTA* T60N (C→A), *TNF* -308 G→A, *AGER* -374 T→A polymorphisms and *HLA-DQB1* risk genotypes as independent and diabetic complication as dependent variable. Age, systolic and diastolic blood pressure, sex, previous/current smoking are included in all models. BMI is included in the models for nephropathy and macrovascular disease, duration in the models for nephropathy and retinopathy and age at diagnosis in the model for macrovascular disease.

Type 1 diabetic patients with diabetic nephropathy had a higher frequency of the *RAGE* -374 A allele (31.2%) compared to those who maintained normoalbuminuria ≥10 years (28.4%) (P = 0.007) (Table 4) and the difference remained even after correction for multiple comparisons (p = 0.02). The allele frequencies of *TNF* -308 G→A and *LTA* T60N polymorphisms were similar in type 1 diabetic patients with and without diabetic nephropathy. No differences in allele or haplotype frequencies of the studied polymorphisms were observed between type 2 diabetic patients with and without diabetic nephropathy (Table 4). In a logistic regression analysis with age, duration, BMI, HbA_1c_, systolic and diastolic blood pressure, sex, previous or current smoking and studied polymorphisms and *HLA-DQB1* risk genotypes as independent variables, having the *LTA* T60N A allele in type 1 diabetic patients was associated with increased risk diabetic nephropathy (OR 2.55[1.11–5.86],p = 0.03), (Figure 1a). In type 2 diabetic patients, the *HLA-DQB1* risk allele but not *LTA*, *TNF* and *AGER* polymorphisms was associated with increased risk for diabetic nephropathy (1.75 [1.05–2.94], p = 0.03). *LTA*, *TNF* and *AGER* polymorphisms were not associated with diabetic nephropathy in type 2 diabetic patients (Figure 1b).

**Table 4:** Allelic association of *LTA* T60N (C→A), *TNF* -308G→A and *AGER* -374 T→A polymorphism with diabetic nephropathy, retinopathy and macrovascular complications.

		Type 1				Type 2			
**NEPHROPATHY**									
		N		p	corrected p	N		p	corrected p
*LTA* T60N C→A	Controls	340	27.9/53.2/18.9	0.317	0.918	442	33.3/47.3/19.5	0.205	0.592
	Cases	113	18.6/63.7/17.7			314	37.2/45.9/16.9		
*TNF* -308 G→A	Controls	342	47.7/46.5/5.8	0.503	0.983	438	63.9/30.4/5.7	0.559	0.970
	Cases	113	44.2/48.7/7.1			314	65.0/30.9/4.1		
*AGER* -374 T→A	Controls	345	52.2/41.2/6.7	0.007	0.023	439	54.2/37.6/8.2	0.119	0.509
	Cases	114	34.2/57.9/7.9			315	58.1/37.1/4.8		
**RETINOPATHY**									
*LTA* T60N C→A	Controls	310	29.4/53.5/17.1	0.154	0.461	584	34.1/49.8/16.1	0.608	0.986
	Cases	310	21.6/60.6/17.7			296	37.5/45.6/16.9		
*TNF* -308 G→A	Controls	307	50.2/43.6/6.2	0.573	0.982	583	65.0/30.7/4.3	0.368	0.878
	Cases	315	46.0/48.9/5.1			295	67.8/28.8/3.4		
*AGER* -374 T→A	Controls	313	48.9/45.3/5.8	0.295	0.774	583	54.9/36.5/8.6	0.734	0.998
	Cases	315	44.8/47.9/7.3			298	50.3/44.0/5.7		
**MACROVASCULAR COMPLICATIONS**									
*LTA* T60N C→A	Controls	609	27.6/55.2/17.2	0.034	0.122	1802	38.1/47.5/14.4	0.102	0.456
	Cases	112	15.2/64.3/20.5			885	35.0/48.6/16.4		
*TNF* -308 G→A	Controls	611	49.3/44.0/6.7	0.426	0.916	1804	67.3/29.2/3.5	0.003	0.017
	Cases	113	42.5/52.2/5.3			886	61.4/34.1/4.5		
*AGER* -374 T→A	Controls	617	48.8/44.6/6.6	0.299	0.797	1809	54.4/38.1/7.5	0.010	0.052
	Cases	111	41.4/52.3/6.3			888	58.8/35.9/5.3		

The uncorrected p-value refers to differences in allele frequencies. Corrected p-value refers to differences in allele frequencies using after 100 000 permutations (Haploview).

### Diabetic retinopathy

The allele (or haplotype) frequencies of the studied polymorphisms did not differ between patients with and without sight-threatening retinopathy, neither in type 1 nor in type 2 diabetic patients (Table 4). In a logistic regression analysis with age, duration, HbA_1c_, systolic and diastolic blood pressure, sex, current/previous smoking and genotypes as independent variables, *HLA-DQB1* risk genotype was associated with decreased risk for sight-threatening retinopathy in type 1 diabetic patients (0.66[0.44–0.99], p = 0.04). In contrast *LTA*, *TNF* and *AGER* polymorphisms were not associated with sight-threatening retinopathy in type 1 diabetic patients (Figure 1a). In type 2 diabetic patients the *AGER* A allele was associated with increased risk for sight-threatening retinopathy (1.65[1.11–2.45], p = 0.01) (Figure 1b).

### Macrovascular complications

Type 1 diabetic patients with a history of macrovascular complications had higher frequency of the *LTA* A allele than patients without macrovascular complications (52.7% vs. 44.8%, p = 0.03) (Table 4). The allele frequencies of the *TNF* and *AGER* polymorphisms did not differ between type 1 diabetic patients with and without history of macrovascular disease.

In a logistic regression analysis, the *AGER* – 374 A allele was associated with increased risk for macrovascular complications (OR 2.05[1.19–3.54], p = 0.01), Figure 1a) in type 1 diabetic patients. The *HLA-DQB1* risk genotype was associated with decreased risk for macrovascular disease in type 1 diabetic patients (OR 0.49[0.28–0.86], p = 0.01). The gene-gene interaction was tested in a separate logistic regression model (assuming a dominant model) by adding a term (AGER)×(HLA risk genotype). The gene-gene interaction term was however not significant and was therefore not included in the final model (Figure 1a).

In type 2 diabetic patients the frequency of both *TNF* -308 A allele and *AGER* -374 T allele genotype was higher in patients with than without macrovascular complications (21.6% vs. 18.1%, p = 0.003 and 76.7% vs. 73.5%, p = 0.03, respectively) (Table 4), the significance of difference in frequency of AGER -374 polymorphism did not stand multiple comparisons (Table 4). The AA haplotype of *TNF* and *LTA* was more common in type 2 diabetic patients with than without macrovascular disease (21.5% vs. 18.1%, p = 0.003). In a logistic regression analysis the *TNF* -308 A allele (OR 1.53[1.04–2.25], p = 0.03) was associated with increased and the *AGER* -374 A allele with decreased risk (OR 0.66[0.49–0.90], p = 0.009) for macrovascular disease (Figure 1b). The gene-gene interaction was tested in a separate logistic regression model (assuming a dominant model) by adding a term (AGER)×(TNF). The gene-gene interaction term was however not significant and was therefore not included in the final model (Figure 1b).

## Discussion

The key finding of the present study was that the polymorphisms in the *TNF*, *LTA* and *AGER* genes were associated with high risk *HLA-DQB1* alleles on chromosome 6p21 and that they influenced the risk for late diabetic complications. The *TNF*, *LTA* and *AGER* alleles were in Hardy-Weinberg equilibrium in as well in non-diabetic controls as in type 2 diabetic patients. In type 1 diabetic patients all of the studied polymorphisms deviated from Hardy-Weinberg equilibrium having excess of heterozygous alleles. This is well in line with previous observations of a synergistic effect of the DR3 and DR4 haplotypes DRB1*0301-DQA1*0501-DQB1*0201 and DRB1*0401-DQA1*0301-DQB1*0302 which are strongly associated with type 1 diabetes thus leading to excess of heterozygous versus homozygous patients [23]. Deviation from Hardy-Weinberg equilibrium will influence the estimated haplotype frequencies especially in a situation with excess of homozygous patients [24]. Therefore, we could not test whether *LTA*, *TNF* and *AGER* polymorphisms are in linkage disequilibrium in type 1 diabetic patients as suggested by Laki et. al. who recently showed that the HLA 8.1 ancestral haplotype (8.1 AH) was strongly linked to the *AGER* -429T→C polymorphism and the AGER -429 allele should therefore be considered as candidate member of the HLA 8.1 ancestral haplotype [22].

The association patterns between diabetic complications and polymorphisms in *LTA*, *TNF*, *AGER* and *HLA* was complex and none of the studied polymorphism was associated with all diabetic complications in either type 1 or type 2 diabetic patients. For example, the A allele of the *AGER* -374 polymorphism was more common in type 1 diabetic patients with than without diabetic nephropathy. In a regression model however, when all of the polymorphisms and *HLA-DQB1* risk genotype were included in the model, *LTA* rather than *AGER* was a risk factor for diabetic nephropathy in type 1 diabetic patients. Similarly, the *AGER* A allele was associated with increased risk for sight-threatening retinopathy but decreased risk for macrovascular disease in type 2 diabetic patients which raises a question, whether this could represent a survival bias because of the strong association between *TNF* and *AGER* polymorphisms and macrovascular disease.

The lack of association in type 1 diabetic patients could of course be due to small sample size. Another possible source of bias could be population stratification due to ethically diverse samples, which is not very likely given the fact that all patients were Scandinavians. Previous studies on the putative association between polymorphisms in the *LTA*, *TNF* and *RAGE* genes and micro-and macrovascular complications in type 2 diabetes have given conflicting results [14]–[20], [25]–[30]. Differences in study design, insufficient power and inclusion of different ethnic groups might explain some of the observed differences, as would inclusion of type 2 diabetic patients with LADA [30]. To circumvent this problem we excluded adult patients who were GAD antibody positive or required insulin therapy during the first year.

Some of the discrepancy in the published literature could also be due to the complex pattern of LD in the region. The HLA region on the short arm of chromosome 6 contains several genes involved in inflammatory responses. The haplotypic structure is complex and there is also a complex interaction between genes and gene products, as illustrated by the *TNF* gene polymorphism that can influence transcription of LTA [8], and receptor for advanced glycation end-products (RAGE), which after binding to its ligand can increase production of pro-inflammatory cytokines, among them TNF-α [31].

Taken together the data show that polymorphisms in the *LTA*, *TNF and AGER* genes increase risk of diabetic micro- and macroangiopathy either alone or together. Given the strong association with HLA-risk genotypes we can not rule out that they are part of a same haplotype and their risk on disease can therefore only be judged from studies assessing them all.

## Materials and Methods

A detailed description of study subjects and analytical methods has been given previously [13]. The study population was mainly the same as in the previous study. However, additional 98 type 1 and 796 type 2 diabetic patients were included and genotyped for the *AGER* -374T→A polymorphism in this study and all the patients were also genotyped for the *TNF* -308 G→A and *LTA* T60N C→A polymorphisms. Patients were classified as having type 1 or type 2 diabetes by the attending physician using the World Health Organization (WHO) guidelines of 1985 [23] or, when diagnosed after January 1, 2001, according to the new WHO guidelines [24]. Type 1 diabetic patients with age at onset >35 years (N = 224) and type 2 diabetic patients positive for GAD antibodies (N = 197) were excluded. In addition, type 2 diabetic patients with an age at diagnosis <35 years and with permanent insulin treatment during the first year from diagnosis were excluded (N = 108). A total of 3699 (742 type 1 and 2957 type 2 diabetes) Scandinavian patients and 206 Scandinavian, non-diabetic control subjects were included in the study. Control subjects were selected among spouses of patients with hypertension; they were not allowed to have any first degree relatives with diabetes. No information on hypertension or myocardial infarction in non-diabetic control subjects was available. The Ethics committee of Malmö/Lund approved the study. Informed consent was obtained from all patients.

### Assessment of complications

#### Diabetic nephropathy

The urinary albumin concentration was determined by immunonephelometry (Beckman Instruments, CA, USA) until 1998 and thereafter by an immunoturbimetric method (Beckman Coulter, Beckman Instruments, CA, USA). Albuminuria was reported either as µg/min (AER), mg/24 hours or as a urinary albumin/creatinine ratio (g/mol). Microalbuminuria was defined as 20–200 µg/min, 30–300 mg/24 hours or 2.0–25 in males and 2.8–25 g/mol in females. For the definition of microalbuminuria we also considered older values given as the urinary albumin concentration measured in a first morning specimen. Values of 30–300 mg/l were considered as microalbuminuria. Values above the upper limit were indicative of macroalbuminuria. Macroalbuminuria was considered present when at least two values above the cut-off limit for macroalbuminuria were recorded. One positive measurement only was considered as macroalbuminuria if the patient thereafter was treated with ACE inhibitors or angiotensin II receptor blockers or if the patient had had persistent microalbuminuria previously. Patients with other kidney diseases were excluded from the analysis. Normoalbuminuria required that all urinary albumin measurements were within the normal range, otherwise the albuminuria status was considered unknown. Duration of albuminuria was calculated from the onset of microalbuminuria when known, or from the latest measurement with no albuminuria. If not known (60% of all the cases with micro- or macroalbuminuria, 39% in type 1 diabetic patients) the duration was calculated from the first registered value of micro- or macroalbuminuria. When calculating the genotype frequencies in patients with normoalbuminuria, only patients with diabetes duration ≥10 years were included.

#### Diabetic Retinopathy

Information about the retinopathy status was available in 3072 patients. Patients were divided into two groups; subjects with no or non-proliferative retinopathy without macular edema and subjects with sight-threatening retinopathy, which included patients with proliferative retinopathy and/or photocoagulation treatment (panretinal and/or focal/grid for macular edema). The duration of sight-threatening retinopathy was defined from the first information of diagnosis or laser treatment. When calculating the genotype frequencies in patients without sight-threatening retinopathy only those with diabetes duration ≥10 years were included.

#### Macrovascular disease

Macrovascular disease was defined as previously diagnosed myocardial infarction, angina pectoris, transitory ischemic attack (TIA), stroke and/or peripheral vascular disease. Information on previous macrovascular disease was available in 93% of the patients.

In logistic regression analysis age at diagnosis was used in stead of duration because the macrovascular disease duration was often unknown and sometimes a decade before the onset of diabetes. Lipid levels were not used in the regression analysis, because according to the clinical guidelines patients with previous episode of myocardial infarction or stroke should be treated with statins. Consequently, current cholesterol levels were lower in patients with previous episode of macrovascular disease and we did not have access to historical data.

### Genotyping

T60N C→A (rs1041981), *TNF* -308 G→A (rs1800629), and *AGER* -374 T→A (rs1800624) polymorphisms were genotyped using the allelic discrimination method on the ABI 7900 instrument (Applied Biosystems, Foster City, CA). A subset of patient (629 type 1 and 1108 type 2 diabetic patients and all control subjects were previously genotyped for *HLA-DQB1* [13]. The genotyping success rates were 99.0% for the *LTA*, 98.7% for the *TNF* and 98.4% for the *AGER* polymorphisms. Re-genotyping was performed in a separate analysis in random samples from those which passed. A total of 226 (*LTA*), 234 (*TNF*) and 225 (*AGER*) patients were re-genotyped with a 100% genotyping concordance rate.

### Statistical analysis

Data are presented as mean±SD or as median and interquartile range [25^th^–75^th^]. Chi-square test was used to test for frequency differences between studied genotypes. To test differences between group means, the Student's two-tailed t-test was used for normally distributed values and Mann-Whitney U-test for non-normally distributed medians. In order to assess factors associated with diabetic nephropathy, retinopathy and macrovascular disease, a multiple logistic regression analysis with forward selection was performed. All data were analysed with a NCSS 2004 (NCSS statistical software, Kaysville, UT, USA). A p-value <0.05 was considered statistically significant. To evaluate putative haplotype blocks, linkage disequilibrium (LD) between the SNPs was analyzed using Haploview 3.32 and D′ values were calculated with 95% confidence intervals (CI) when the genotype frequencies were in Hardy-Weinberg equilibrium [32]. A corrected p-value was obtained after 100,000 permutations of individual SNPs and haplotype blocks including the *TNF*, *LTA* and *AGER* polymorphisms. Power analysis was made using Genetic Power calculator [33]. HW-QuickCheck software [34] was used for testing of putative excess of heterozygous/ homozygous patients.

#### Power calculations

Power assuming α = 0.05 and relative risk of 1.3 was 11%, 31% and 32% for type 1 diabetic patients and 62%, 81% and 80% for type 2 diabetic patients with or without diabetic nephropathy for the *LTA*, *TNF* and *AGER* polymorphisms. The power for retinopathy was 68%, 86% and 85% in type 1 and 58%, 73% and 73% in type 2 diabetes and for macrovascular disease 20%, 28% and 28% in type 1 and 97%, 99% and 99% in type 2 diabetes.
